# Stochastic losses of fire‐dependent endemic herbs revealed by a 65‐year chronosequence of dispersal‐limited woody plant encroachment

**DOI:** 10.1002/ece3.3020

**Published:** 2017-05-10

**Authors:** John Stephen Brewer

**Affiliations:** ^1^ Department of BiologyUniversity of MississippiUniversityMSUSA

**Keywords:** beta‐diversity, endemism, fire exclusion, floristic quality, neutral theory, niche, phylogenetic diversity, Raup–Crick distance, taxonomic distinctness

## Abstract

The factors responsible for maintaining diverse groundcover plant communities of high conservation value in frequently burned wet pine savannas are poorly understood. While most management involves manipulating extrinsic factors important in maintaining species diversity (e.g., fire regimes), most ecological theory (e.g., niche theory and neutral theory) examines how traits exhibited by the species promote species coexistence. Furthermore, although many ecologists focus on processes that maintain local species diversity, conservation biologists have argued that other indices (e.g., phylogenetic diversity) are better for evaluating assemblages in terms of their conservation value. I used a null model that employed beta‐diversity calculations based on Raup–Crick distances to test for deterministic herbaceous species losses associated with a 65‐year chronosequence of woody species encroachment within each of three localities. I quantified conservation value of assemblages by measuring taxonomic distinctness, endemism, and floristic quality of plots with and without woody encroachment. Reductions in herb species richness per plot attributable to woody encroachment were largely stochastic, as indicated by a lack of change in the mean or variance in beta‐diversity caused by woody encroachment in the savannas studied here. Taxonomic distinctness, endemism, and floristic quality (when summed across all species) were all greater in areas that had not experienced woody encroachment. However, when corrected for local species richness, only average endemism and floristic quality of assemblages inclusive of herbs and woody plants were greater in areas that had not experienced woody encroachment, due to the more restricted ranges and habitat requirements of herbs. Results suggest that frequent fires maintain diverse assemblages of fire‐dependent herb species endemic to the region. The stochastic loss of plant species, irrespective of their taxonomic distinctness, to woody encroachment suggests that the relevance of niche partitioning or phylogenetic diversity to the management of biodiversity in wet pine savannas is minimal.

## 1 Introduction

Management to maintain biodiversity requires understanding the factors that maintain species coexistence and produce communities of high conservation value. Management activities are often aimed at manipulating extrinsic factors (e.g., disturbance regimes) that promote short‐term species coexistence. For example, many of the world's most species‐rich plant communities at small sampling grains (<50 m^2^) are seminatural temperate grasslands maintained by frequent mowing, burning, or chronic grazing (Wilson, Peet, Dengler, & Pärtel, 2012). The extraordinarily high species richness of semidry grasslands of the Czech Republic, with up to 44 species per 0.25 m^2^ (Klimeš, Dančak, Hájek, Jongepierová, & Kučera, 2001), is thought to be the result of thousands of years of chronic grazing or mowing (Hájková et al., 2011; Wilson et al., 2012). Such chronic low‐intensity disturbance prevents competitive displacement of small herbs by larger herbs and woody plants (Klimeš et al., 2001). Such hyperdiverse assemblages appear to support hypotheses of disturbance‐mediated species coexistence (e.g., Huston, 1979) and pose a challenge for niche theory predictions that there are over 40 different niches per 0.25 m^2^ in these communities (Wilson et al., 2012).

In contrast to most management perspectives on preserving diversity, most ecological theory is concerned with how traits exhibited by the species themselves promote species coexistence (Chesson, 1991). Disturbances and/or low rates of competitive displacement, although potentially and often important in maintaining species coexistence in the short term (Connell, 1978; Hubbell, 2001; Huston, 1979, 2014), are argued not to be sufficient for long‐term species coexistence (Chesson, 2000; Fox, 2013; but see Huston, 2014). For this reason, several ecologists have argued that niche differences or density‐dependent stabilizing mechanisms are necessary to explain the maintenance of species diversity over the long term, even when competitive equivalence and disturbances are sufficient to maintain short‐term species coexistence (Chesson, 2000; Fox, 2013). Indeed, some empirical studies of plant communities appear to corroborate the prediction (e.g., Adler, Ellner, & Levine, 2010; Harms, Wright, Calderón, Hernández, & Herre, 2000; Levine & HilleRisLambers, 2009). Importantly, there have been few attempts to test the importance of deterministic species coexistence based on species differences in hyperdiverse plant communities that have been chronically disturbed for millennia [and thus perhaps unlikely to exhibit long‐term stability (Myers & Harms, 2009)].

Notwithstanding the inordinate focus of ecologists on processes that promote local species diversity, several conservation biologists have argued that local species diversity does not adequately quantify the conservation value of assemblages (Faith, 1992; May, 1990; Vane‐Wright, Humphries, & Williams, 1991). Some conservation biologists have argued that preserving phylogenetic diversity of assemblages is of the utmost importance in preserving conservation value of assemblages (Faith, 1992). In contrast to species diversity, phylogenetic diversity accounts for the evolutionary uniqueness of the species that comprise the assemblage (Faith, 1992). Protecting assemblages of taxonomically distinct species may be more effective at preserving functional diversity than is protecting assemblages of less taxonomically distinct species (Faith, 1992; Vane‐Wright et al., 1991; Vellend, Cornwell, Magnuson‐Ford, & Mooers, 2011). Hence, management that results in losses of, say, multiple congeneric species may result in less of a loss of biodiversity than management that results in losses of, say, multiple genera or families. Other conservation biologists have argued in favor of considering how disturbance of the regional landscape has diminished the fraction of endemic species or species indicative of globally rare habitats within local assemblages (Bibby, 1994; Brewer, 2008; Faith, Reid, & Hunter, 2004; Noss, 1990). Population declines and local extinctions of these globally rare species contribute more to the global biodiversity crisis than do losses of globally common species (Bibby, 1994; Pimm et al., 2014; Silva et al., 2015; Thomas, 2013; Vellend et al., 2013). Shared traits among these species that allow them to tolerate extreme local abiotic conditions such as chronic/very frequent fires, but that also prevent them from competing effectively with less tolerant species, could contribute to their restriction to such rare, chronically disturbed habitats and thus their endemism.

Most land managers and ecologists agree that the high plant species richness found in longleaf pine (*Pinus palustris*) communities (e.g., up to 42 species per 0.25‐m^2^; Walker & Peet, 1984) is maintained in large part by frequent fires (Folkerts, 1982; Gilliam & Christensen, 1986; Glitzenstein, Streng, Masters, Robertson, & Hermann, 2012; Glitzenstein, Streng, & Wade, 2003; Palmquist, Peet, & Weakley, 2014; Provencher et al., 2001; Walker & Peet, 1984). In cases where fire frequencies have been reduced in pine savannas, species diversity of herbaceous plants often declines (Glitzenstein et al., 2003, 2012; Palmquist et al., 2014). Increased competition with woody plants and to a lesser extent large grasses appears to be responsible for much of the loss of herbaceous species in longleaf pine savannas (Brockway & Lewis, 1997; Glitzenstein et al., 2003, 2012; Hinman & Brewer, 2007; Myers & Harms, 2009; Palmquist et al., 2014).

What is less clear is to what extent niche differences contribute to the extraordinarily high species diversity found in frequently burned longleaf pine savannas (Brewer, 2006; Myers & Harms, 2009, 2011). Niche differences among herbs (e.g., differences in flowering seasons, dispersal syndromes, and mechanisms of nutrient uptake) may also be necessary to maintain species coexistence of herbs in pine savannas (Brewer, 2003; Myers & Harms, 2009; Platt, Evans, & Davis, 1988; Walker & Peet, 1984). If so, then fire exclusion could also result in the disproportionate loss of particular herb species that occupy the same niche as woody species or cause the stochastic displacement by woody species of co‐occurring herb species that occupy complementary niches. In other words, both frequent fires and ecologically important differences among species are necessary for coexistence of fire‐dependent herbs. A null hypothesis is that competitive (fitness) equivalence and dispersal limitation allow local stochastic coexistence of herbaceous species (Hubbell, 2001; Myers & Harms, 2009). Hence, as long as fires occur frequently enough to prevent competitively dominant woody species from establishing and displacing herbs, the more‐or‐less competitively equal and subordinate herbaceous species will persist via stochastic processes.

Little is known about whether declines in species richness with fire exclusion disproportionately result from losses of taxonomically distinct species, rare endemics, and/or pine savanna specialists. A previous study of the effects of invasion of mesic longleaf pine savanna by a nonnative invasive grass, *Imperata cylindrica*, revealed that dramatic losses in groundcover species coincided with comparable reductions in percent endemism and fidelity to pine savannas (Brewer, 2008). The effect of native woody encroachment during fire exclusion on these measures of conservation value, however, has not been quantified. Furthermore, to my knowledge, reductions in phylogenetic diversity or distinctness in response to invasions or woody encroachment has not been investigated for pine savannas. Given that different measures of conservation value may give different results (Daru, van der Bank, & Davies, 2015), it seems prudent to examine a variety of measures.

The objectives of the current study were twofold. First, to elucidate the factors responsible for maintaining diverse herbaceous groundcover plant communities in three frequently burned pine savannas in southern Mississippi, I used a null model to investigate deterministic (nonrandom) species losses associated with long‐term woody species encroachment. I hypothesized that deterministic losses of herb species with woody encroachment would indicate functional differences among coexisting herbs that could be important in their coexistence. The null hypothesis was that competitive equivalence of herb species in relation to woody species would result in stochastic losses of herb species with woody encroachment. Second, to elucidate factors important in maintaining high conservation value of groundcover plant communities in these savannas, I measured taxonomic distinctness, endemism, and fidelity of assemblages to relatively pristine wet pine savannas (floristic quality) in sampling plots with and without woody encroachment. I hypothesized that all three measures of diversity would decline with declines in species richness, but that each may or may not decline with woody encroachment after correcting for local species richness. If local species richness is, by itself, not an adequate metric for evaluating assemblages in terms of their conservation value, then species composition of assemblages must be evaluated in terms of conservation value in a manner not confounded with species richness.

## 2 Methods

### 2.1 Study sites and sampling design

Data for the current study come from measurements of plant species richness and composition in 1997 at three sites in Desoto National Forest in Stone County, Mississippi, USA. The three sites (hereafter Sandy Creek, Wolf Branch, and Little Red Creek) contained open wet longleaf pine savannas. Poor drainage, low pH, and periodic fires resulted in a grass‐sedge dominated community. *Pinus elliottii* (slash pine) invaded most savannas in the region following logging and fire exclusion in the 1900s (Harper, 1914; Heyward, 1939). It was the dominant overstory species at all three sites in 1997. Slash pine stems greater than 1.5 m tall serve as effective perches for animal‐dispersed seeds of trees, shrubs, and vines (Hinman, Brewer, & Ashley, 2008). As a result, dense woody thickets form at the base of pines, which have substantially lower plant species richness due to losses of herbaceous species (Brewer, 1998a). Greater seedling establishment of woody species beneath pines is not related to more favorable environmental differences near pines, but rather is limited by seed dispersal (Brewer, 2002; Hinman et al., 2008). From the early 1980s to 1996, the three sites were burned once every 3 years in the winter, which was effective at preventing additional establishment by slash pine and other tree species. As a result, there were open areas away from pines dominated by grasses, sedges and pitcher plants and clearly defined thickets of shrubs, small trees, and vines clustered around slash pine trees (Brewer, 2002). Prior to sampling in 1997, Wolf Branch was last burned in January of 1996, Sandy Creek was last burned in November of 1996, and Little Red Creek was last burned in January of 1995.

To quantify differences in plant species richness and composition between open grass‐sedge areas and areas near pines, I sampled 16 plots (0.25 m × 0.25 m) located within a ~ 0.5‐ha open portion of the savanna at each site. Each plot was located greater than 5 m from the closest slash pine tree. In addition, 16, 16, and 14 plots, respectively, were established within woody thickets adjacent to (i.e., within 1 m) a slash pine greater than 5 cm diameter‐at‐breast‐height (dbh) at Wolf Branch, Little Red Creek, and Sandy Creek in the general vicinity (within 20 m) of the plots in the open areas. I determined the stage of woody encroachment and reduction in plant species richness within patches using a chronosequence, that is, by coring and aging pines associated with plots. Censuses at all three sites were conducted in May and September of 1997. Results of both censuses were combined into a single response, which included the presence/absence of each species encountered during the season in which it was most likely to be encountered and identified (e.g., when flowering).

### 2.2 Deterministic losses of herb species during a chronosequence of woody encroachment

**Figure 1:**
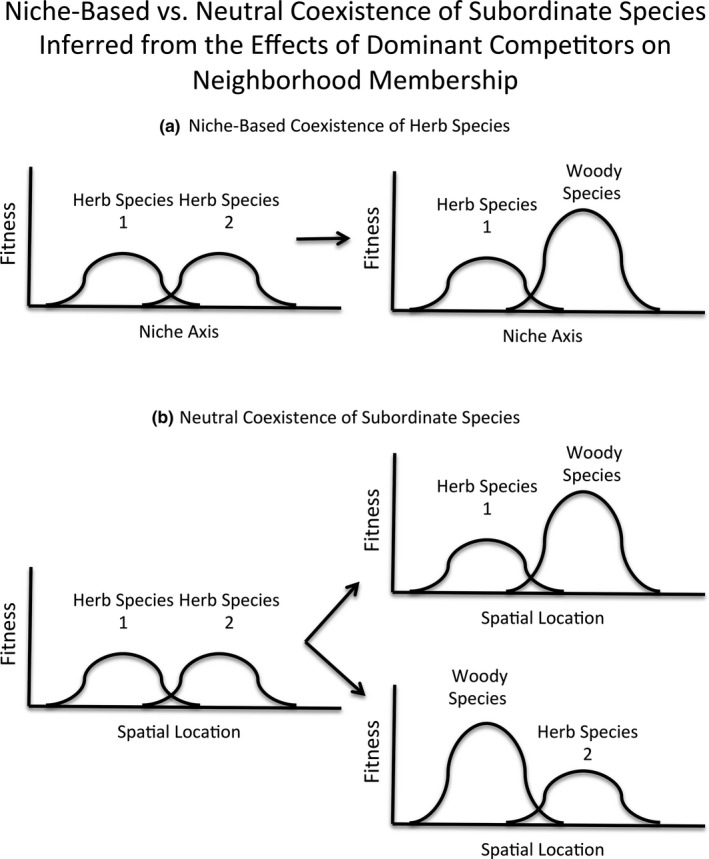
Predicted patterns of competitive displacement of herbs by encroaching dominant woody plants in wet pine savannas. If coexistence of herbs is the result of (a) niche differences and superior woody competitors occupy the same niche as some herbs (in this example, herb species 2) but not others, then displacement will be deterministic, leaving herbaceous assemblages that are more similar to one another than expected by chance. If coexistence of herbs is the result of (b) stochastic processes and each herb species competes equally poorly with superior woody competitors, then displacement will be stochastic, leaving herbaceous assemblages that are no more similar to one another than expected by chance

To assess whether deterministic factors contributed to species losses associated with long‐term woody species encroachment, I used an approach that examines the effects of dominant competitors on species composition of assemblages of subordinate species (see also Myers & Harms, 2009). Specifically, I calculated one aspect of beta‐diversity (i.e., variation in species composition, Anderson et al., 2011; Whittaker, 1972) among neighborhoods within or away from woody thickets that developed during a prior period of up to 65 years of fire exclusion. Assuming that dominant competitors (i.e., woody species) dramatically reduced species richness of subordinate herb species within sampling plots (i.e., reduced alpha‐diversity; see Brewer, 1998a; for support), a significant reduction in beta‐diversity (reduced variation in composition among plots) would indicate that woody competitors deterministically eliminated herbaceous species from their neighborhoods. The resulting assemblages near trees would be more similar to one another in composition than would assemblages in open areas away from trees. Beta‐diversity could decline near trees if certain herb species were consistently displaced by competitively superior woody plants that occupied the same niche (Figure 1). Alternatively, if the herb species losses associated with tree and shrub establishment were stochastic (the null prediction), then herbaceous beta‐diversity of areas near trees should be similar to that in open areas. Competitive displacement of certain herb species would be just as likely as competitive displacement of other herb species (Figure 1). Such a response would suggest that most herbs were competitively equivalent and thus functionally similar with respect to their ability to compete with woody plants. Even if most or all herbs were equally poor competitors compared to woody plants, certain herb species might co‐occur more often than expected by chance as a result of each species occupying a different niche (i.e., allowed combinations, sensu Diamond, 1975) or responding more or less equally well to particular environmental conditions at those locations in which they co‐occur (Tilman, 2004). Such losses would not necessarily result in mean differences in beta‐diversity between thickets and open areas, but they should result in greater variance in beta‐diversity within thickets than in open areas (see Appendix S1). Accordingly, I examined differences in the variance in beta‐diversity between patch types.

### 2.3 Declines in conservation value of groundcover assemblages with woody encroachment

To quantify taxonomic distinctness, endemism, and floristic quality of plot assemblages, I first calculated values of taxonomic distinctness at the genus level and species‐specific values of endemism and fidelity to wet pine savannas that lack severe anthropogenic disturbance and then either summed or averaged these values across all species within each sampling plot. Summed or averaged taxonomic distinctness provided measures of phylogenetic diversity, whereas summed or averaged values of endemism and fidelity to wet pine savannas provided assemblage‐level values of endemism and floristic quality, respectively.

### 2.4 Taxonomic distinctness

**Figure 2:**
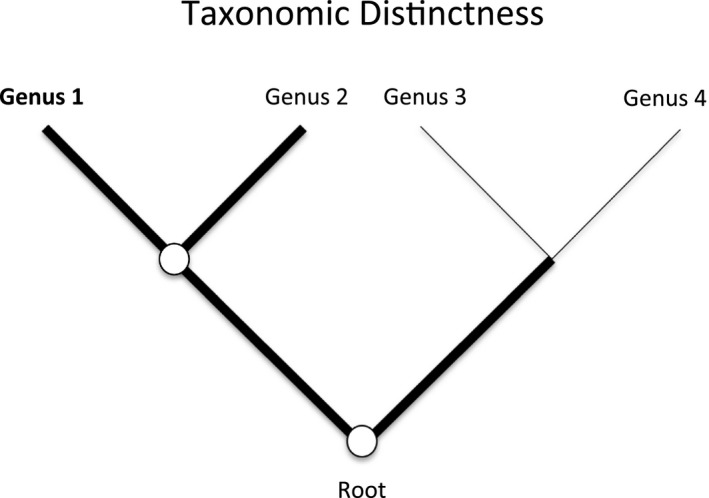
Calculation of taxonomic distinctness of genera encountered. I identified all nodes from the family containing the genus in question (Genus 1 in this example) to the root of land plants (2 nodes in this oversimplified example) and then counted the sum of all branches at these nodes (4 in this example). I calculated taxonomic distinctness for each genus by taking the inverse of the number of branches for each genus (1/4 in this example). In those cases with soft unresolved polytomies of many genera, I calculated the expected number of branches from the genus to the family root using the formula, 2 × log_2_ (# of genera). In a perfectly balanced binary tree (as shown in the example), the number of nodes (down to and including the node at the family root) containing a genus is log_2_ (# of genera within the family). Assuming each node produces only two descendants, the total number of branches is 2 × # of nodes

I first calculated values of taxonomic distinctness at the genus level and then summed these values across all species within each sampling plot. I used May's (1990) modification of methods developed by Vane‐Wright et al. (1991). Using the Tree of Life Web Project phylogenetic tree for land plants and the most recently published Angiosperm Phylogeny Group phylogeny (APG IV 2016), I identified all nodes from the family containing the genus in question to the root of land plants and then counted the sum of all branches at these nodes (Figure 2). To quantify the number of nodes and branches from the genus in question to the family root, I used a variety of data sources from the primary literature (Appendix 1). In those cases with soft unresolved polytomies of many genera, I calculated the expected number of branches from the genus to the family root using the formula, 2 × log_2_ (# of genera). In a perfectly balanced binary tree, the number of nodes containing a genus is log_2_ (# of genera). Assuming each node produces only two descendants, the total number of branches is 2 × # of nodes (Figure 2). I calculated taxonomic distinctness for each genus by taking the inverse of the number of branches for each genus. Taxonomic distinctness of each plot was quantified by calculating taxonomic richness (sum of the taxonomic distinctness values for all species in a plot; WS_TD_) and by calculating weighted mean taxonomic distinctness (WM_TD_).

### 2.5 Fidelity to wet pine savannas (Floristic quality)

Floristic quality was calculated using a different approach than that originally proposed by Swink and Wilhelm (36). Using a list of 454 species I had encountered in field plots throughout Mississippi (see also Brewer & Bailey, 2014), I generated a species by habitat matrix by consulting several regional flora manuals (Appendix S2) and recording the habitats in which each species occurred. I then subjected the habitats to a Bray–Curtis ordination, in which axis 1 endpoints were subjectively chosen such that anthropogenically disturbed habitats represented one end of the axis and wet pine savannas (including bogs) represented the other end of the axis (see McCune & Grace, 2002; for further description of this ordination technique). I then used the resulting axis 1 scores for the habitats to calculate weighted averages for the species to obtain species scores. Species scores ranged between 0 and 1, with higher scores indicating association with wet pine savannas and lower scores indicating association with anthropogenically disturbed areas. I used the resulting species scores to calculate weighted sums (summed floristic quality; WS_FQ_) and weighted averages of presence/absence for each sampling plot in the current study (WM_FQ_). These weighted averages were comparable to a measure of floristic quality (weighted mean fidelity to wet pine savannas as opposed to anthropogenically disturbed habitats). All species encountered in the wet pine savannas studied here were native. Bray–Curtis ordination was made using PC‐Ord version 6 (McCune & Grace, 2002).

### 2.6 Endemism

Endemism was quantified by consulting the USDA Plants database maps for each species in the dataset and then counting the number of states or commonwealths in the USA (including Puerto Rico) and the number of provinces in Canada in which the species occurred. For those species with distributions that extended into south Texas, I used alternative sources to determine whether species also occurred in Mexico or Central America. I then took the inverse of these counts. High values therefore indicated strong geographic restriction to just a few states in the southern USA. Low values indicated widespread distributions and occurrence throughout eastern North America and beyond. I then used the resulting endemism values for each species to calculate weighted sums (summed endemic diversity; WS_E_) and weighted averages of presence/absence for each sampling plot in the current study (WM_E_).

### 2.7 Statistical analysis

I contrasted beta‐diversity of neighborhoods associated with slash pine/shrub thickets to those in open areas away from thickets in each of the three wet pine savanna sites using a permutation‐based dispersion test (Permdisp, Anderson, 2006). I did a separate analysis for each site. Permdisp tests were conducted for statistically significant differences between groups with respect to the mean compositional distance from each group's centroid. To ensure that differences in multivariate dispersion near versus away from trees were not simply the result of differences in alpha‐diversity, I calculated Raup–Crick distances using the raupcrick function in R (package vegan; Chase, Kraft, Smith, Vellend, & Inouye, 2011). Unlike other distance or dissimilarity measures (e.g., Bray–Curtis, Jaccard, Euclidean), Raup–Crick distances are based on deviations from expected dissimilarities derived from a null model that accounts for the expected relationship between beta‐diversity and alpha‐diversity (Anderson et al., 2011; Chase et al., 2011). Deterministic losses of species due to woody encroachment were indicated by significantly lower mean Raup–Crick distance from the group centroid in tree‐centered thickets than in open areas. Differences in compositional dispersion between tree‐associated thickets and open areas were depicted graphically using a nonmetric multidimensional scaling (NMS) ordination based on Raup–Crick distances. To test the null hypothesis that the loss of each individual herb species was independent of losses of any other species, I used Permdisp to quantify the variance in beta‐diversity (i.e., the mean Euclidean distance in Raup–Crick deviations from the group centroid of deviations) in thickets and in open areas. A rejection of the null hypothesis could result from the variance in beta‐diversity being greater in thickets than in open areas due to losses of *combinations* of herb species that were observed more or less often than expected by chance. Permdisp and NMS were both conducted using Primer 6 plus PERMANOVA (Anderson, Gorley, & Clarke, 2008).

Each conservation value response variable (WS_TD_, WM_TD_, WS_FQ_, WM_FQ_, W_SE_, W_ME_) was subjected to a two‐way analysis of variance, in which the effects of site (Wolf Branch, Sandy Creek, Little Red Creek), encroachment (open areas vs. tree‐associated thickets), and their interaction were tested using the residual error term. For each response variable, I conducted an analysis using all groundcover plant species and another using herbaceous species only. I did two‐way ANOVA analyses using the *lm* function in R.

## 3 Results

### 3.1 Stochastic losses of herbs with woody encroachment

**Figure 3:**
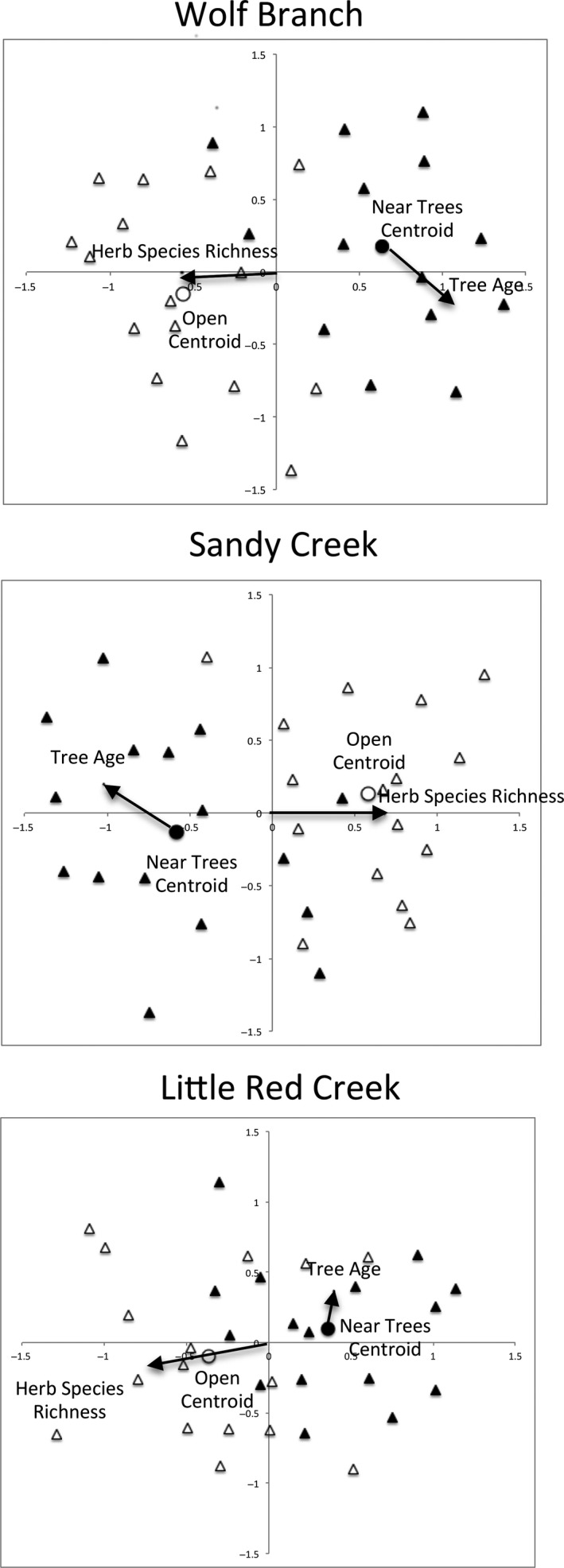
Nonmetric multidimensional scaling ordination of sampling plots in herb species space (based on Raup–Crick distances) showing overlain vectors of herb species richness near (filled triangles) and away from (open triangles) tree‐centered thickets of varying ages in three wet pine savannas in southeastern Mississippi

Despite reductions in alpha species richness near trees (Figure 3), in no savanna did beta‐diversity differ significantly between tree/shrub thickets and open areas away from thickets (Wolf Branch pseudo‐*F*_1,28_ = 0.019; *p* = .91; *n* = 14 and 16 for thickets and open areas, respectively; Sandy Creek pseudo‐*F*_1,30_ = 0.43; *p* = .49; *n* = 16; Little Red Creek pseudo‐*F*_1,30_ = 0.72; *p* = .41; *n* = 16; Figure 3). Furthermore, herb species richness declined dramatically with age of woody encroachment at all three sites, but such declines were not associated with significant declines in beta‐diversity at any of the three sites (Table 1). Hence, the results suggest that trees and/or associated shrubs stochastically displaced herb species from their neighborhoods. Variance in beta‐diversity was not greater in thickets than in open areas at any of the three sites (Wolf Branch pseudo‐*F*_1,209_ = 0.31; *p* = .61; Sandy Creek pseudo‐*F*_1,238_ = 0.06; *p* = .81; Little Red Creek pseudo‐*F*_1,238_ = 0.57; *p* = .52). Hence, there was no evidence of stochastic losses of allowed or favored combinations of herb species. The loss of each individual species associated with woody encroachment appeared to be independent of that of other species.

**Table 1:** Relationships between herb species richness and age of woody thicket (# of growth rings in slash pine at thicket center) and herbaceous beta‐diversity (quantified as Raup–Crick distance of sample from centroid of woody thickets in herb species space)

Site	Pearson correlation, *r*, of herb species richness with age of woody thicket (*p* value in parentheses)	Pearson correlation of distance from centroid with herb species richness in woody thickets (*p* value in parentheses)
Wolf branch	−.58 (.03)	−.20 (.46)
Sandy creek	−.90 (≪.01)	.30 (.26)
Little red creek	−.67 (<.01)	.12 (.66)

### 3.2 Losses of taxonomic distinctness, floristic quality, and endemism with woody encroachment

**Figure 4:**
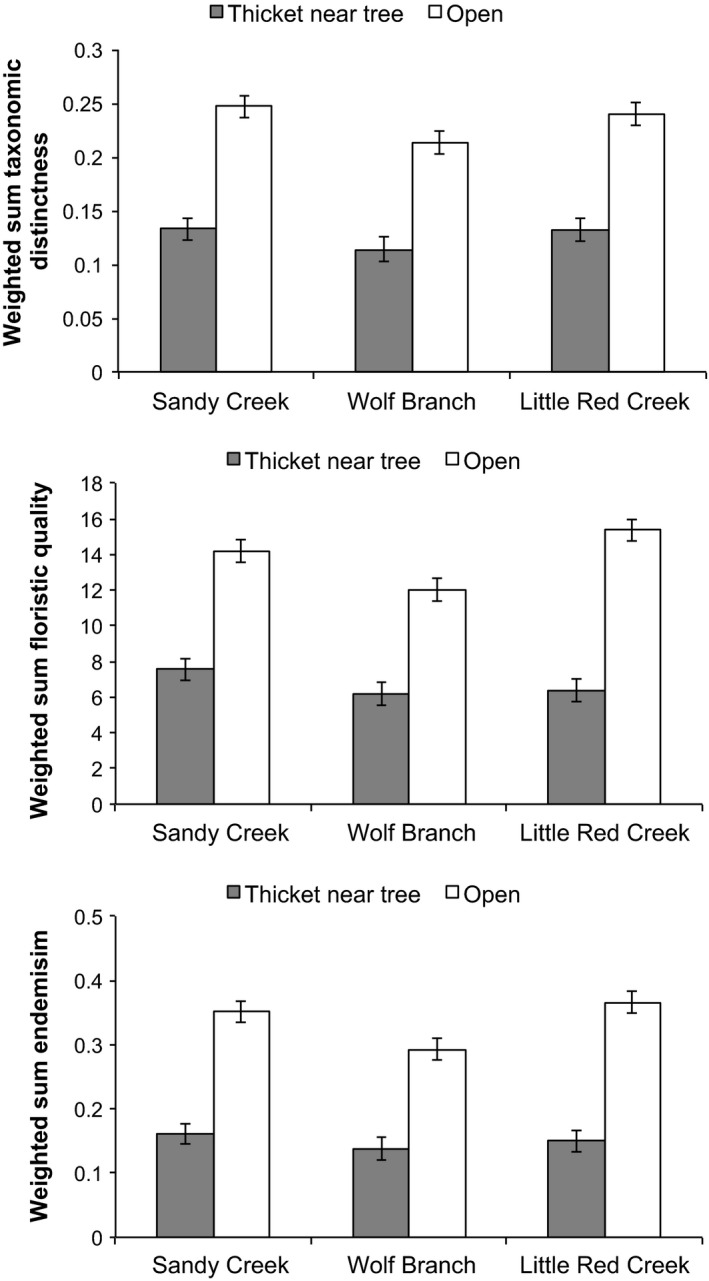
Weighted summed taxonomic distinctness, floristic quality, and endemism of sampling plots in open areas and in thickets near trees at three sites based on herbaceous species presence/absence data. Values are least‐squares means ± 1 *SE*. Overall mean difference in taxonomic distinctness for location relative to trees was 0.11; overall *SE* = 0.01 and 0.01 for open areas and thickets, respectively; overall sample size = 48 and 46 for open areas and thickets, respectively. Overall mean difference in floristic quality for location relative to trees was 7.14; overall *SE* = 0.62 and 0.63 for open areas and thickets, respectively. Overall mean difference in endemism for location relative to trees was 0.19; overall *SE* = 0.02 and 0.02 for open areas and thickets, respectively

When summed across all species in each plot, taxonomic distinctness (WS_TD_), floristic quality (WS_FQ_), and endemism (WS_E_) were all greater in areas that had not experienced woody encroachment (Figure 4). Such responses largely reflected the highly positive correlations between these weighted sums and species richness (*r* > .89). Considering all groundcover plants and herbs alone, there was a highly significant effect of encroachment on WS_TD_ (*F*_1,88_ = 77.07, *p* ≪ .01; *F*_1,88_ = 156.06, *p* ≪ .01, respectively; there were a total of 46 plots in thickets and 48 plots away from trees). The effect of site approached statistical significance (*p* = .06 and .07, respectively), but the site × encroachment interaction was not significant (*p* = .49 and .79, respectively), the latter indicating that the negative effect of encroachment on WS_TD_ was consistent among sites. There was a highly significant effect of encroachment on WS_FQ_ (*F*_1,88_ = 144.95, *p* ≪ .01; *F*_1,88_ = 196.08, *p* ≪ .01, for all groundcover species and herbs alone, respectively). WS_FQ_ differed significantly among sites (*F*_2,88_ = 6.36, *p* < .01; *F*_2,88_ = 4.11, *p* < .01, for all groundcover species and herbs alone, respectively; Figure 4). The site × encroachment interaction was statistically significant (*p* = .05 and .03, respectively), but WS_FQ_ was significantly higher in open areas than near trees at all three sites (Tukey's honest significant difference >5.93; *p* ≪ .01; Figure 3). There was a highly significant effect of encroachment on WS_E_ (*F*_1,88_ = 138.88, *p* ≪ .01; *F*_1,88_ = 192.52, *p* ≪ .01, for all groundcover species and herbs alone, respectively). WS_E_ differed significantly among sites (*F*_2,88_ = 4.28, *p* = .05; *F*_2,88_ = 3.18, *p* = .02, for all groundcover species and herbs alone, respectively), but the site × encroachment interaction was not significant (*p* = .18 for both; Figure 4).

### 3.3 Changes in average taxonomic distinctness, floristic quality, and endemism with woody encroachment

**Figure 5:**
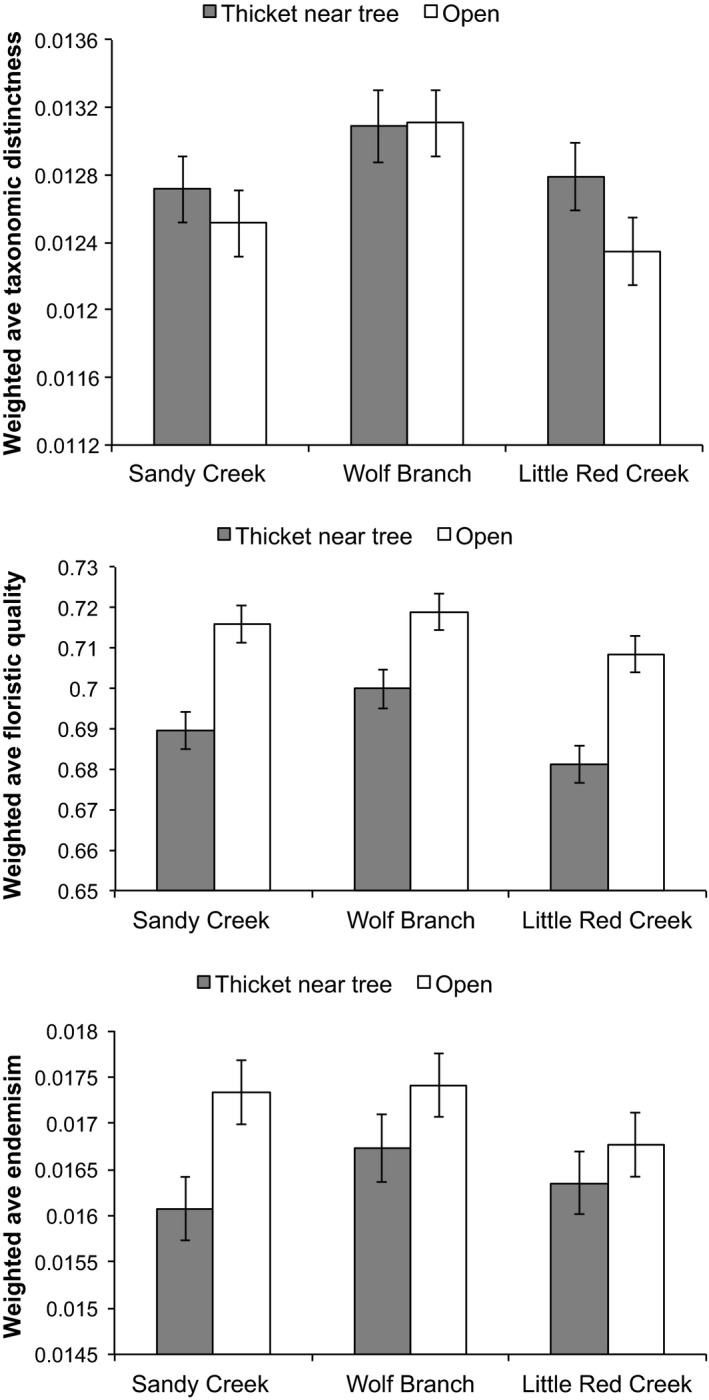
Weighted averages of taxonomic distinctness, floristic quality, and endemism of sampling plots in open areas and in thickets near trees at three sites based on the presence/absence of all groundcover species (herbaceous and woody). Values are least‐squares means ± 1 *SE*. Overall mean difference in taxonomic distinctness for location relative to trees was 0.00021; overall *SE* = 0.00020 and 0.00020 for open areas and thickets, respectively; overall sample size = 48 and 46 for open areas and thickets, respectively. Overall mean difference in floristic quality for location relative to trees was 0.024; overall *SE* = 0.0046 and 0.0047 for open areas and thickets, respectively. Overall mean difference in endemism for location relative to trees was 0.00078; overall *SE* = 0.00034 and 0.00035 for open areas and thickets, respectively

**Figure 6:**
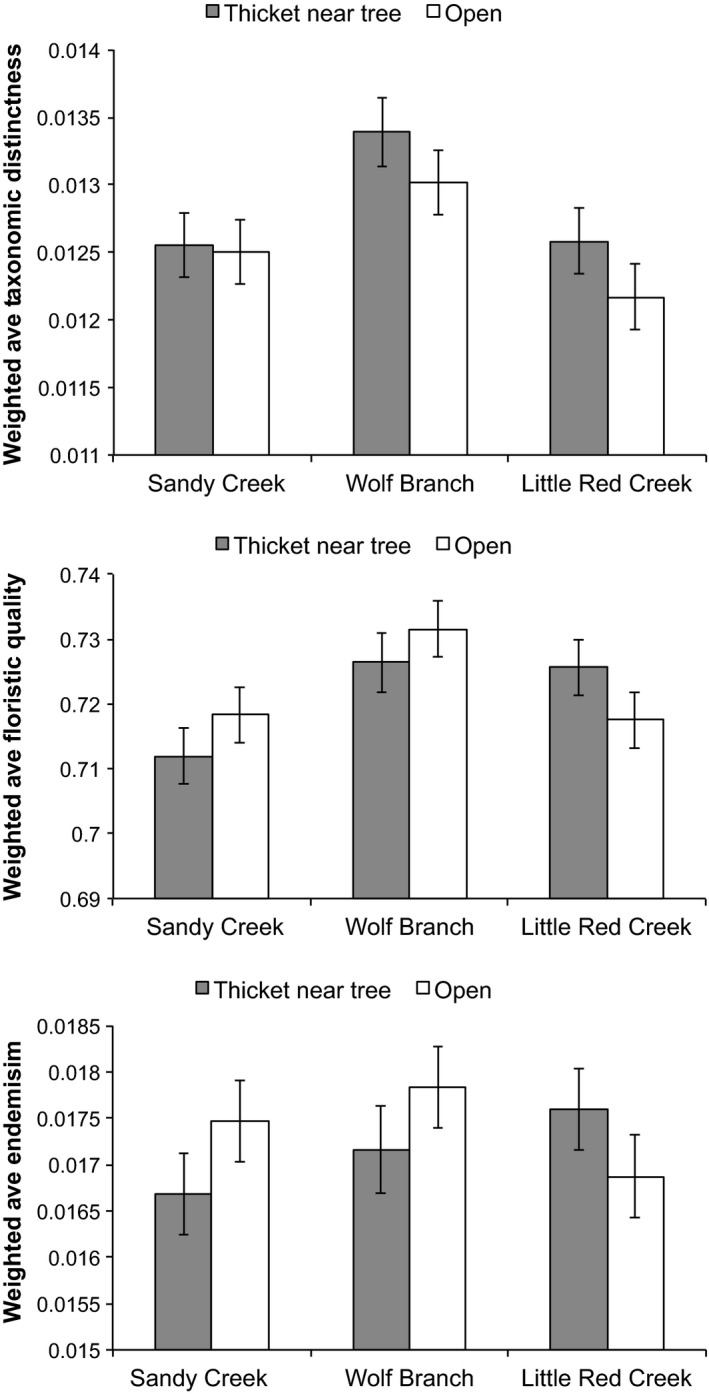
Weighted averages of taxonomic distinctness, floristic quality, and endemism of sampling plots in open areas and in thickets near trees at three sites based on the presence/absence of herbaceous species. Values are least‐squares means ± 1 *SE*. Overall mean difference in taxonomic distinctness for location relative to trees was 0.00028; overall *SE* = 0.00024 and 0.00025 for open areas and thickets, respectively; overall sample size = 48 and 46 for open areas and thickets, respectively. Overall mean difference in floristic quality for location relative to trees was 0.0011; overall *SE* = 0.0043 and 0.0044 for open areas and thickets, respectively. Overall mean difference in endemism for location relative to trees was 0.00024; overall *SE* = 0.00044 and 0.00045 for open areas and thickets, respectively

In contrast to the responses of summed measures of taxonomic distinctness, endemism, and floristic quality, only average endemism and floristic quality of plot assemblages (including herbs and woody species) were greater in areas that had not experienced woody encroachment (Figure 5). Considering all groundcover plants and herbs alone, there was no effect of encroachment on WM_TD_ (*p* = .19; *p* = .17, respectively; Figures 5 and 6). Weighted mean taxonomic distinctness of plot assemblages differed significantly among sites (*F*_2,88_ = 4.21, *p* = .02; *F*_2,88_ = 6.25, *p* < .01, for all groundcover species and herbs alone, respectively; Figures 5 and 6). The site × encroachment interaction, however, was not significant (*p* = .52 and .73, respectively), the latter indicating that site differences in WM_TD_ were not affected by woody encroachment.

When all groundcover species were considered (i.e., herbs and woody plants), weighted mean floristic quality of plot assemblages was lower within thickets than in open areas away from pines, as indicated by a highly significant effect of encroachment on WM_FQ_ (*F*_1,88_ = 41.75, *p* ≪ .01; Figure 5). The same effect was not observed when considering herbs alone (*p* = .77; Figure 6). Hence, stochastic losses of herb species reduced floristic quality because herbs were more indicative of wet pine savannas than were woody species. WM_FQ_ differed significantly among sites (*F*_2,88_ = 5.39 and 5.13, *p* < .01, for all species and herbs alone, respectively). The site × encroachment interaction was not significant, regardless of whether all groundcover species or just herbs were considered (*p* = .63 and .18, respectively; Figures 5 and 6).

When all groundcover species were considered, weighted mean endemism of plot assemblages was lower in thickets than in open areas away from pines, as indicated by a highly significant effect of encroachment on WM_E_ (*F*_1,88_ = 7.70, *p* < .01; Figure 5). The same effect was not observed when considering herbs alone (*F*_1,88_ = 0.40, *p* = .53; Figure 6). Hence, as with floristic quality, stochastic losses of herbs reduced average endemism because herbs were more likely to be endemic to the southeastern USA than were woody species. WM_E_ did not differ significantly between sites (*p* = 0.29 and 0.61, for all groundcover species and herbs alone). The site × encroachment interaction was not significant (*p* = .46 and .18, for all groundcover plants and for herbs alone, respectively).

## 4 Discussion

Results of this study highlight the importance of reduced competition from woody plants and cast doubt on the importance of niches in maintaining species diversity in hyperdiverse savannas of the southeastern USA. Consistent with a prediction of neutral theory (Hubbell, 2001), the current study showed that the establishment and development of tree‐centered thickets apparently resulted in stochastic losses of herbaceous groundcover plant species in three diverse wet pine savannas. Two pieces of evidence support the conclusion of stochastic species loss. First, the reductions in alpha (local) richness did not result in reductions in beta‐diversity, as would be expected if losses of certain species were greater than those of other species (Chase et al., 2011). Competitively subordinate herb species thus did not differ in their ability to compete with dominant woody competitors. Second, despite as much as a 90% reduction in species richness in some species‐rich plots, in which many species were represented by a single individual, the variance in beta‐diversity did not increase with woody encroachment. Hence, woody encroachment did not result in stochastic losses of favored combinations of multiple species.

Because the development of shrub thickets in this study was associated with established pine trees, it is not possible to distinguish the effects of shrubs from those of the pines on herbs in this study. Frequent fires have previously been shown to reduce the abundance and size of shrubs, which in turn may benefit some herb species more than others (Palmquist et al., 2014). In the system studied here, the pine trees, themselves, rather than the associated shrubs may be primarily responsible for the stochastic losses of herbs. As with many of the herb species studied here, *Drosera capillaris* was more abundant away from pine trees (Brewer, 1998a), due in part to higher seedling emergence and survival of adults away from pines (Brewer, 1998b). Nevertheless, the effect of killing shrub‐dominated groundcover near trees on seedling emergence and transplant survival was no greater than the effect of killing herb‐dominated groundcover vegetation away from trees (Brewer, 1998b).

The chronosequence used here is not a perfect substitute for long‐term experiments, and thus, caution is warranted when interpreting the results. Furthermore, the results do not mean that density‐dependent stabilizing mechanisms are absent in this system (Chesson, 1991). Negative feedbacks associated with host‐specific natural enemies could promote species coexistence among herbaceous species in the absence of woody encroachment (Bever, 1994; Connell, 1971; Janzen, 1970; Reynolds, Packer, Bever, & Clay, 2003). Long‐term monitoring of populations of herbaceous species to determine whether each species is able to increase when rare in a neighborhood is needed to test such a hypothesis (Adler et al., 2010).

The use of null models based on presence/absence beta‐diversity to test for niche vs. neutral models of community structure has been criticized recently (Tucker, Shoemaker, Davies, Nemergut, & Melbourne, 2016). The approach may not always be sensitive enough to distinguish between purely stochastic processes and assembly processes that incorporate both stochasticity and niche differences (Tucker et al., 2016). Hence, I cannot completely discount the possibility that niche differences among established herbs contributed to their initial coexistence, and the current approach is not a perfect substitute for a detailed analysis of functional traits. In the current study, however, I examined losses within “old growth” groundcover communities subjected to fire exclusion, rather than assembly of developing communities. Hence, even if niche differences played some role in community assembly, such differences appeared to play little or no role in the “disassembly” of established groundcover plant communities subjected to fire exclusion. Fine‐scale niche differences between established plants are therefore probably irrelevant to fire‐related diversity management of established groundcover communities in this system.

Whereas taxonomic distinctness, endemism, and floristic quality, when summed across all species in each plot, were all greater in areas that had not experienced woody encroachment, when averaged across all species in a plot, only endemism and floristic quality were greater in areas that had not experienced woody encroachment. Hence, for the system studied here, endemism and floristic quality were more informative measures of conservation value than was taxonomic distinctness. The reduction in average endemism and floristic quality resulted from the fact that herbs were more likely to be associated with wet pine savannas and endemic to the southeastern US Coastal Plain than were woody species and the former were disproportionately lost to woody encroachment. Fire exclusion therefore reduces the regional distinctness of pine savanna vegetation as a result of stochastic losses of endemic herbs and dispersal‐limited gains of widespread woody plants. Because endemic herbaceous species (as opposed to more widespread woody species) were disproportionately affected, continued fire exclusion that leads to additional woody encroachment potentially could contribute to a generally acknowledged decline in global biodiversity by reducing the population sizes and thus increasing the likelihood of extinction of endemic herbs (Pimm et al., 2014; Thomas, 2013; Vellend et al., 2013).

The negative effect of fire exclusion and woody encroachment on a taxonomically diverse array of endemic plant species indicative of fire‐dependent pine savannas is consistent with a hypothesis of convergent adaptation of pine savanna herbs to frequent fires in the southeastern USA. Frequent fires in fact reduce the presence of one functional group: woody species. It is the exclusion or reduction of this particular functional group that helps to maintain species diversity, endemism, and floristic quality by preventing competitive exclusion (Brewer, 1998a; Glitzenstein et al., 2003; Hinman & Brewer, 2007; Hinman et al., 2008; Myers & Harms, 2009; Palmquist et al., 2014). As a group, fire‐tolerant herbs are clearly functionally diverse and include species that differ with respect to photosynthetic pathway, dispersal syndrome, mycorrhizal associations, nitrogen fixation, reliance on carnivory, hemi‐ or holo‐parasitism, and flowering and growth phenology (Palmquist et al., 2014; Walker & Peet, 1984). How such trait differences contribute to species coexistence, however, is unclear. So, the question remains what could maintain species diversity over the longer term. One possible explanation is increased diversification (speciation) associated with fire tolerance. The evolution of fire‐stimulated flowering in goldenasters (*Pityopsis*) was found to be associated with a greater incidence of sympatric or geographically localized speciation within the fire‐prone southeastern USA (Gowe & Brewer, 2005; Teoh, 2009). Hence, the same factor that allows for reduced extinction (frequent fires) might also contribute to increased diversification.

Regardless of which factors are responsible for long‐term species coexistence, the results of the current study suggest that land managers should focus on managing fire regimes in wet pine savannas in such a way as to minimize competitive exclusion of herbaceous species. Such fire regimes are likely to also promote phylogenetic diversity, endemic diversity, and the frequency of species specialized to survive in frequently burned pine savannas.

## Conflict of Interest

None declared.
