# Supplementary material for: Stochastic losses of fire‐dependent endemic herbs revealed by a 65‐year chronosequence of dispersal‐limited woody plant encroachment
Source: Ecol Evol. 2017 May 10;7(12):4377–89. doi: 10.1002/ece3.3020 (PMC5478063; doi:10.1002/ece3.3020)
Supplement: Supplementary file 1 [file ECE3-7-4377-s001.docx]

Appendix S1. Demonstration of increased variance in beta-diversity resulting from stochastic losses of favored combinations of species compared to stochastic losses of individual species.

First, calculate the number of random combinations, unordered without replacement, of a given number of species that survive establishment by woody plants, *X_i(ind)_*, given a certain number of species that were present in a patch before establishment by woody plants using the formula for the binomial coefficient:

$X_{i(ind)}=\frac{s_{initial}!}{\left( s_{survivors}! \right)\left( s_{initial}-s_{survivors} \right)!}$ (1)

Where, *s_initial_* is the number of herb species in a patch before invasion by woody plants and *s_survivors_* is the number of herb species in the same patch after invasion by woody plants, with the constraint that 0 < *s_survivors_* < *s_initial_*.

Second, calculate the number of random combinations, unordered without replacement, of a given number of favored combinations of species that survived establishment by woody plants, *X_i(fav)_*, given a certain number of favored combinations of species that were present in a patch before establishment by woody plants:

$X_{i(fav)}=\frac{c_{initial}!}{\left( c_{survivors}! \right)\left( c_{initial}-c_{survivors} \right)!}$ (2)

Where, *c_initial_* is the number of favored combinations of herb species, unordered, without replacement, in a patch before invasion by woody plants and *c_survivors_* is the number of favored combinations herb species in the same patch after invasion by woody plants, with the constraint that 0 < *c_survivors_* < *c_initial_*.

If favored or allowed combinations are, by definition, groups containing more than one species, where the expected number of co-occurring species in each group equals the quantity, *f*, and we assume that

$c_{initial}=\frac{s_{initial}}{f}$ (3)

then *s_initial_* > *c_initial_*. If we also assume that the fraction of species loss for an area in which the loss of individual species, *l_(ind)_*, is random, is the same as that in an area in which the loss of favored combinations of species, *l_(fav)_*, is random, such that

$l_{(ind)}=1-\frac{s_{survivors}}{s_{initial}}= l_{\left( fav \right)}= 1-\frac{c_{survivors}}{c_{initial}}$ (4)

we can prove (see Figure A1) that

$X_{i(ind)}> X_{i(fav)}$ (5)

Figure A1. Predicted number of possible combinations (*X_i(ind)_* and *X_i(fav)_*, respectively) for a situation in which one-half of the number initial herb species (*S_initial_*) or of the initial number of favored combinations of herb species (C_initial_) survives woody encroachment of a pine savanna. The number of species within each favored combination is assumed to be 2; therefore, *C_initial_* is assumed to be ½ of *S_initial_*. The possible combinations considered are assumed to be unordered and without replacement.

Accordingly, given that losses of species or favored combinations of species are random, such that all possible surviving species combinations are equally likely in a given area, the probability of a given surviving species combination occurring in a given area is equivalent to the inverse of the number of possible surviving combinations in each area:

$\frac{1}{X_{i(ind)}}< \frac{1}{X_{i(fav)}}$. (6)

Let

$\frac{1}{X_{i(ind)}}=P_{i(ind)}$ and $\frac{1}{X_{i(fav)}}=P_{i(fav)}$ (7)

and thus,

$P_{i(fav)}>P_{i(ind)}$. (8)

Accordingly, for a given species combination, *i*, the probabilities that another patch will have the same species combination for the area with favored combinations of species and the area with random combinations of individual species, respectively, are:

$P_{i(fav)}^{2}; P_{i(ind)}^{2}$ (9)

Hence, for the area with favored combinations of species, the overall probability that any two patches will contain the same combination of species is

${{P_{same\left( fav \right)}= X}_{i(fav)}P}_{i(fav)}^{2}$ (10)

and, for the area with random combinations of species, the overall probability that any two patches will contain the same combination of species is

${{P_{same\left( ind \right)}= X}_{i(ind)}P}_{i(ind)}^{2}$ (11)

Likewise, the overall probabilities that two patches will share no species in common for the area with favored combinations of species and for the area that contains random assemblages of species, respectively, are:

${{P_{none shared\left( fav \right)}= X}_{i(fav)}P}_{i(fav)}^{2}$ (12)

and

${{P_{none shared\left( ind \right)}= X}_{i\left( ind \right)}P}_{i(ind)}^{2}$ (13)

Substituting the inverse probabilities from equation 7 for *X_i(fav_*_)_ and *X_i(ind)_* in equations 10 through 13 and simplifying, we find that the probabilities that two patches will have the exact same species composition or share no species in common is equivalent to the probabilities that a given species combination will occur by chance, such that:

${{P_{same\left( fav \right)}= P}_{none shared\left( fav \right)}=P}_{i(fav)}$ (14)

and

${{P_{same\left( ind \right)}= P}_{none shared\left( ind \right)}=P}_{i(ind)}$ (15)

Hence, because $P_{i(fav)}>P_{i(ind)}$, we find that

$P_{same(fav)}>P_{same(ind)}$ (16)

and

$P_{none shared(fav)}>P_{none shared(ind)}$ (17)

Compared to the area with random combinations of species, in the area with favored combinations of species, a greater fraction of the pairs of patches will have proportional (dis)similarities near the extremes (1 or 0), and a smaller fraction of the pairs of patches will have proportional (dis)similarities near the average (0.5). Therefore, the frequency distribution of dissimilarities among patches in the area with favored combinations of species will have greater breadth (variance) and will have fatter distribution tails (platykurtic) than will the frequency distribution of dissimilarities among patches in the area with random combinations of individual species.

The variances in beta-diversity for the area with favored combinations and the area with random combinations of species, respectively, are

$\sigma_{beta(fav)}^{2}= \sum[\left( D_{i\left( fav \right)}-\bar{D} \right)^{2}\times\frac{1}{N_{D}}]$ (18)

and

$\sigma_{beta(ind)}^{2}= \sum[\left( D_{i\left( fav \right)}-\bar{D} \right)^{2}\times\frac{1}{N_{D}}]$ (19)

Where *σ^2^* is the variance in beta-diversity, *D_i_* is the proportional dissimilarity between two given patches, $\bar{D}$ is the mean dissimilarity among patches and *N_D_* is the number of dissimilarities considered. Variance increases as *P_i_* and thus as *P_same_* (where *D_i_* = 0) and *P_none shared_* (where *D_i_* = 1) increase. Because *P_i(fav)_* > *P_i(ind)_*, assuming the same number of patches are compared between areas, *σ^2^_beta(fav)_* > *σ^2^_beta(ind)_*_._

The current study contrasts *σ^2^_beta_* of groups of patches (plots) of herb assemblages that have lost species to woody encroachment with *σ^2^_beta_* of groups of patches that have not lost species to woody encroachment. Raup-Crick distances were used to calculate both mean and variance in beta-diversity within groups so as to correct for the association of these moments with alpha-richness (patch species richness). Random and independent losses of individual species should not result in increased variance in beta-diversity if based on Raup-Crick distances, whereas random losses of significant combinations of species, as demonstrated above, should.
